# Horizontal Gene Transfers in prokaryotes show differential preferences for metabolic and translational genes

**DOI:** 10.1186/1471-2148-9-9

**Published:** 2009-01-10

**Authors:** Aditi Kanhere, Martin Vingron

**Affiliations:** 1 Max Planck Institute for Molecular Genetics, Ihnestraße 63–73, 14195 Berlin, Germany; 2 MRC/UCL Centre for Medical Molecular Virology, Division of Infection and Immunity, University College London, London W1T 4JF, UK

## Abstract

**Background:**

Horizontal gene transfer (HGT) is an important process, which contributes in bacterial pathogenesis and drug resistance. A number of methods have been proposed for detection of horizontal gene transfer. One successful approach to the detection of HGT events is due to Novichkov *et al*. (J. Bacteriology 186, 6575–85), who rely on comparing phylogenetic distances within a gene family with genomic distances of the source organisms. Building on their approach, we introduce outlier detection in the correlation between those two sets of distances. This approach is designed to detect horizontal transfers of core set of genes present in many bacteria. The principle behind method allows detection of xenologous gene displacements as well as acquisition of novel genes.

**Results:**

Simulations indicated that our method performs better than Novichkov *et al*'s original approach. The approach very efficiently identified HGT between distantly related bacteria and also a limited number of gene transfers between closely related bacteria. In combination with sequence similarity and likelihood tests, it yields a measure robust enough to derive a set of 171 genes deemed likely to have been horizontally transferred. Further analysis of these 171 established horizontal transfer events gave interesting insights in the direction of transfer.

**Conclusion:**

The majority of transfers between archaea and bacteria have occurred in the direction from bacteria to archaea rather than the other way round. Genes transferred between the archaea and bacteria are mostly metabolic genes. On the other hand, genes transferred within the bacterial phyla are mainly involved in translation.

## Background

Transfer and subsequent incorporation of genetic material from one organism to a phylogenetically distinct organism is termed *Horizontal Gene Transfer *(HGT). Horizontally transferred genes are thought to confer biological properties, which are beneficial to the host organism. It is also often seen that a horizontally transferred gene provides the recipient organism better compatibility with its ecological environment. One of the examples of such ecological adaptation is the presence of horizontally acquired archaeal genes in the thermophilic bacteria like *Aquifex aeolicus *[1]. Bacteria are also known to acquire antibiotic resistance as well as virulence properties via HGT [2]. Horizontal gene transfer thus contributes to bacterial evolution and possibly plays an important role in speciation [3].

A number of methods have been proposed to detect horizontally transferred genes. These can be broadly classified as nucleotide compositional methods, phylogenetic methods and similarity based methods. The basic idea behind compositional methods is that each genome has a compositional signature [4-6] and hence, genes acquired from foreign genomes are expected to be distinct in their composition from the rest of the host genome. Phylogenetic methods, on the other hand, rely on comparison of a gene tree with a reference tree and subsequent detection of incoherence [3,6,7]. Similarity-based methods generally use BLAST based searches to find the most related sequence [1,8,9]. If this closest Blast hit is from a distant taxon, then the sequence is identified as horizontally transferred.

Compositional methods are computationally less demanding than phylogenetic methods. They require only knowledge of the genome under study. The fundamental problem with compositional methods is the assumption that the same genomic signature can be applied to all parts of the genome, thus possibly mistaking compositionally biased proteins like membrane proteins or ribosomal proteins for horizontally transferred genes. In addition, these methods are unable to detect transfer among compositionally similar organisms. It is also often difficult to determine the source of a horizontally transferred gene using compositional methods.

The phylogenetic methods are most rigorous and probably most accurate but are computationally more demanding. It is very difficult to automate tree comparison methods. Hence, most of the phylogenetic studies on horizontal gene transfer are focused on few genomes or few gene families.

Although similarity based methods are computationally less demanding, these methods have been criticized because the closest BLAST hit does not necessarily imply phylogenetic proximity [10]. The strength of their predictions depends on the sequence similarity criteria used. It has been also reported that genome size and variability in rates of evolution can affect results of these methods [11]. Like compositional methods, on their own, sequence similarity methods cannot detect the direction of transfer, either. In addition, some of the similarity based methods like the one described by Podell and Gaasterland [8] require knowledge of a whole proteome. As observed by these authors, such an approach can lead to false positives in case of insufficient database coverage of the given species. It has been shown that the HGT predictions by different methods have very little overlap [12].

**Figure 1:**
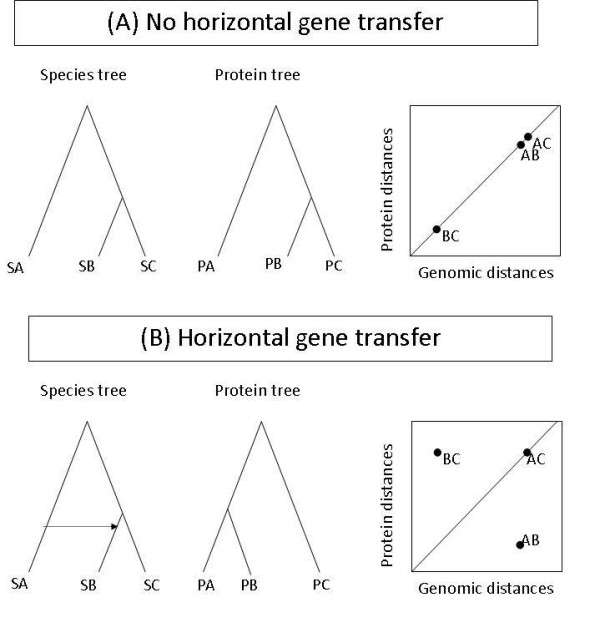
**Schematic diagram of changes in protein distances with HGT**. (A) In a protein family where no horizontal transfer of gene has occurred, the protein tree will exactly follow species tree. In such a case, plotting genomic distances vs. protein distances will show a perfect correlation. (B) On the other hand, if a gene is horizontally transferred, e.g., from species SA to species SB, the protein tree will not resemble the species tree. In this case, some protein distances (like BC) will appear to be greater and some other protein distances (like AB) will appear to be smaller than the corresponding genomic distances. These outlying points (BC, AB) will result in disturbance in correlation and can be detected with Cook's distance.

Novichkov *et al*. proposed a robust approach for detecting HGTs which involves neither building phylogenetic trees nor compositional analysis [13]. This approach is based on comparing evolutionary distances within a set of orthologs to corresponding intergenomic distances (Figure 1). In the absence of HGT and under uniform evolutionary rates, one expects that evolutionary distances between orthologs and corresponding intergenomic distances display a linear relationship (Figure 1A). A gene transfer between a donor species and an acceptor species, however, will result in outliers (Fig. 1B). Novichkov *et al*. used multiple statistical tests to rank genes according to their probability to have been horizontally transferred. They applied this method to selected genomes and to selected groups of orthologous proteins (Clusters of Orthologous Groups of proteins, COGs), which share same function [14]. This approach was designed to detect xenologous gene displacement (XGD), in which a gene is displaced by an ortholog from different lineage.

Here, we simplify Novichkov *et al*.'s approach by introducing one single measure, Cook's distance [15,16], to detect HGT. This simplification helps us to apply the method to a much larger set of genomes and a much larger number of COGs than analyzed by Novichkov *et al*. Using simulation we demonstrate that our method can detect horizontal transfer at much closer distance than Novichkov *et al*.'s method. Combining this approach with sequence similarity we can also detect the direction of HGT.

In this study, we analyzed 1965 orthologous protein families from the COG database [see Additional file 1]. Combining the proposed protocol with stringent sequence similarity search and likelihood tests, we have also built a dataset of 171 genes, which, with high confidence, have undergone horizontal transfers. Our analysis of these 171 HGTs between different prokaryotic domains gives interesting insights into the direction of transfers as well as functional characteristics of transferred genes. We find that most of the transfers between archaea and bacteria have occurred from bacteria to archaea. The majority of genes transferred between archaea and bacteria are involved in metabolism, while most of the transfers within the bacterial domain involve translational genes.

## Results

As previously mentioned, in the absence of HGT, distances among proteins, evolving at constant rate, should be proportional to the respective genomic distances (Fig. 1A). A gene transfer between a donor species and an acceptor species, however, will result in individual protein distances that disagree with the corresponding genomic distances (Fig. 1B). Such transferred genes can thus be detected as outliers. After analyzing several outlier detection measures, we found that Cook's distance (CDISS) performed well for outlier detection. As described in Methods, we used an average CDISS value, <CDISS>, for all the distances involving a gene under study. In this paper, we used this approach to analyze HGT in large number of proteins families.

For this analysis, we relied on Clusters of Orthologous Groups of proteins or COGs [14]. The orthologous groups in COG database were built by automated sequence searches, followed by careful manual curation for functional conservation. We further filtered COGs to retain only 1965 groups, which show reasonable correlation between inter-protein and intergenomic distances. These measures were taken to ensure a constant rate of evolution.

Although there are various ways to calculate intergenomic distances, none of them is without limitation. In this analysis, we utilized 16S rRNA sequences for this calculation. It needs to be mentioned that rare cases of horizontal transfer of ribosomal genes are known [17,18]. However, availability and analysis of a large number of prokaryotic genomes have shown that evolutionary relationships among prokaryotes are generally well represented by 16S rRNA sequences [19,20].

### Simulation and assessment of <CDISS> measure

**Figure 2:**
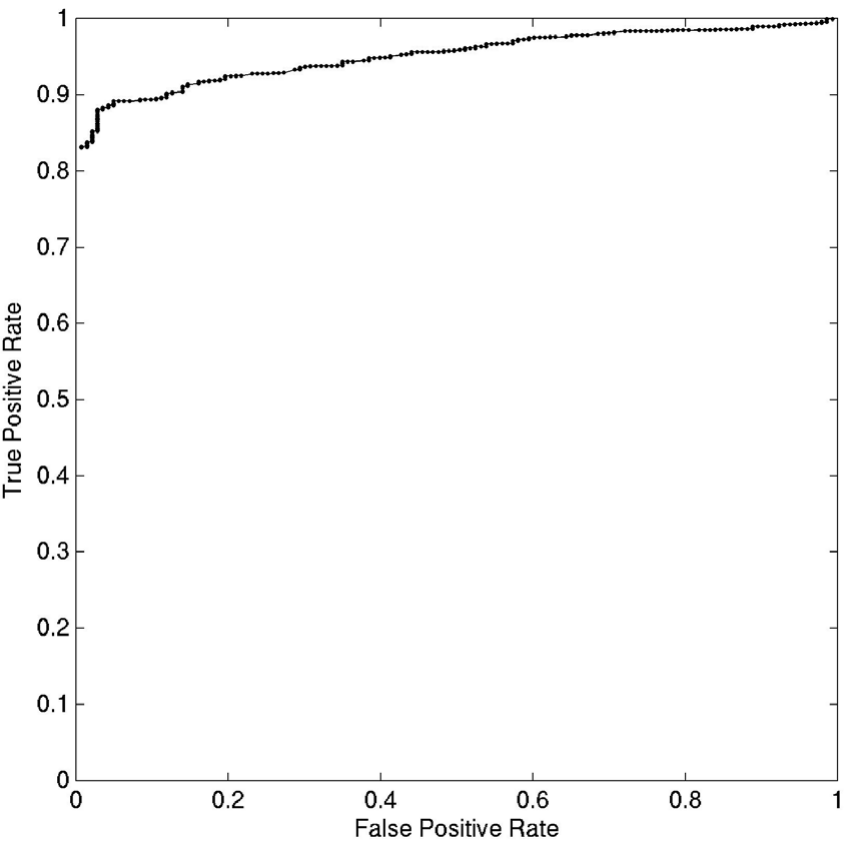
**ROC curve analysis carried out on simulated data**. As described in the text, false positive accumulation rate and true positive accumulation rate were calculated by varying the <CDISS> cut-off at regular intervals. The false positive and true positive rates at different <CDISS> cut-offs are plotted.

In order to assess the performance of CDISS in detecting HGT, we first carried out an *in silico *experiment. This simulation was performed on 8 COG families (COG1660, COG1666, COG1949, COG2844, COG3091, COG3852, COG4536, COG5007), which showed very high correlation (r >0.95 and p-val < 10^-5^) between rRNA and protein distances. The correlation as well as the visual inspection of the protein trees indicated that the phylogenies of these families were very close to the species trees as derived from 16S rRNA sequences. To simulate a HGT scenario for a COG family, we randomly selected an acceptor and a donor species. We replaced the protein-protein distances involving acceptor species with corresponding distances involving donor species. The procedure was carried out 100 times on each of the above 8 COGs. In each case, the <CDISS> value corresponding to the horizontally transferred sequence was noted. Figure 2 shows an ROC curve for this simulation at various cut-offs on <CDISS>. For further calculations, the <CDISS> cut-off corresponding to 5% false positives was chosen. At this cut-off, 90% of true positives could be detected. It should be noted that a realistic scenario, where the transferred gene can diverge due to various cellular processes such as amelioration, can be much more complicated and difficult to detect. On the other hand, the protein sequences, which passed this stringent cut-off, are very likely to have originated from another organism.

**Figure 3:**
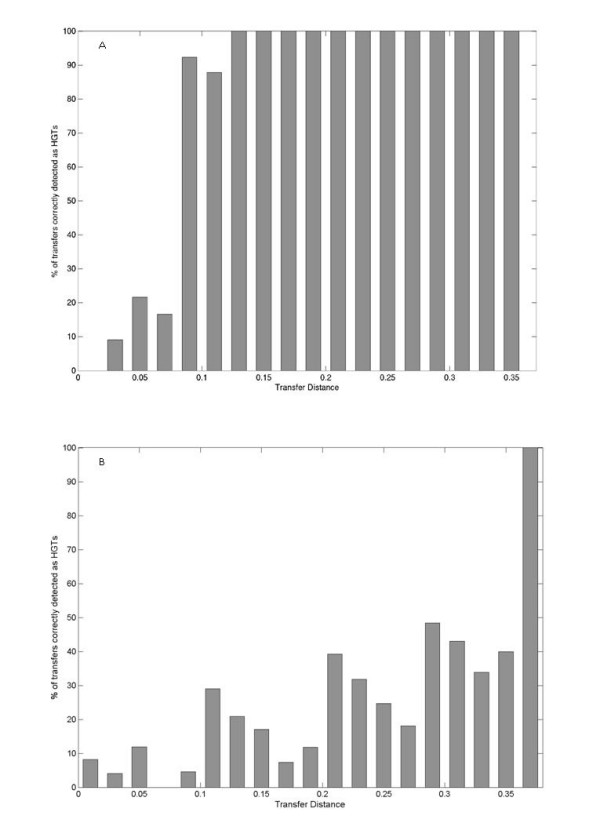
**The percentage of correctly detected HGT events at different transfer distances**. A) This analysis is carried on simulated data as described in text. It can be seen that detection of HGT increases as the distance between acceptor and donor species increases. The detection reaches 100% when the transfer distance is greater than 0.12. But ~40% of horizontally transferred genes can still be detected at lower transfer distances (16S rRNA < 0.1). B) Similar simulation was carried out using Novichkov *et al*.'s method. It can be seen that Novichkov et al.'s method is customized to detect only transfers between very distantly related bacteria (16S rRNA > 0.36).

Acquisition of a gene from a distant bacterium can result in stronger disturbances in protein distances as compared to gene transfer between closely related species. It is hence easier to detect distant transfers compared to gene transfers between closer species. To evaluate the sensitivity of the method, it is important to see the distribution of genomic distances between acceptor and donor species in our predictions. Figure 3A shows the percentage of correctly predicted HGT events at various transfer distances. It can be seen that although the frequency of HGT detected at closer transfer distances was low, the method could detect a number of transfers between closely related species. We could correctly detect around 40% of the transfers between species, which are closer than a 16S rRNA distance of 0.1 nucleotide substitutions per site. In the ideal case where the correlation between 16S rRNA and protein distance was perfect (r = 1.0), the method picked up all the simulated transfers, independent of the distance.

For comparison, we also tested the approach of Novichkov *et al*. using this simulation. Again, we calculated the number of correctly predicted HGTs in simulated transfers. Figure 3B shows the percentage of correctly predicted HGTs at various transfer distances by their approach. Using Novichkov *et al*. approach, smaller number of HGTs was detected when compared to our method. Since their method was designed to predict only XGDs, simulated transfers only at a 16S rRNA distance > 0.36 nucleotide substitutions per site could be identified.

### Detection of HGT in aminoacyl-tRNA synthetase families

The aminoacyl-tRNA synthetases are fundamental for the process of translation. Many groups have studied the phylogenetic organization of these proteins [21-23]. The phylogenetic trees of aminoacyl-tRNA synthetases show various anomalies as compared to the species tree. These anomalies have been explained by many genetic events like gene duplication, domain acquisition, loss of genes as well as horizontal gene transfer. In recent years, horizontal gene transfer in some of these molecules has been studied in detail [21,22,24-26]. These aminoacyl-tRNA synthetases constitute a good example to test the proposed method.

Table 1 lists aminoacyl-tRNA synthetases for which there is strong evidence supporting horizontal gene transfer. It is interesting to see that our method predicted 6 out of 8 cases correctly. Among the remaining 2 cases, HGT in the histidyl-tRNA synthetase family would have been predicted with slightly relaxed cut-off of 1.3/D.

**Table 1:** Previously reported HGT among aminoacyl-tRNA synthetases

Name of Synthetase^*^	Horizontal transfer	COG	Our Predictions	References	Extra predictions (Protein IDs)^Ψ^
Leucyl-tRNA sythetase	Bacteria to Halobacterium	COG0495	Y	17	MJ0633, SSO0589, aq_1770, aq_351
Phenylanalyl-tRNA sythetase	Archaea to Spirochaetes	COG0016	Y	18	MA0171
Prolyl-tRNA sythetase	Archaea to Deinococcus, Borrelia and Mycoplasma	COG0442	Y	16,18	ML1553
Valyl-tRNA sythetase	Archaea to Rickettsia	COG0525	Y	13	RSp0782^Θ^
Histidyl-tRNA sythetase	Archaea to Helicobacter, C. acetobutylicum, Spirochaetes	COG0124	N	18	TM0143
Lysyl-tRNA sythetase	Archaea to Spirochaetes	COG1384	Y	21	---
Cysteinyl-tRNA sythetase	Bacteria to Methanosarcina, Archaeoglobus	COG0215	N	18	ML1302, MYPU_1770_2
Seryl-tRNA sythetase	Bacteria to Halobacterium	COG0172	Y	18, 19	AGc4663, MA4048, MJ1077, MK1460, mll1081

^*^ Only those cases, for which there is a clear evidence of horizontal gene transfer and which are present in our databset, are shown.

^Ψ ^We predict that 14 more proteins in these families may have undergone horizontal gene transfer. However, there is no literature reference for these predictions.

^Θ^ RSp0782 is probably a false positive. Only small part of this sequence is included in this family.

**Figure 4:**
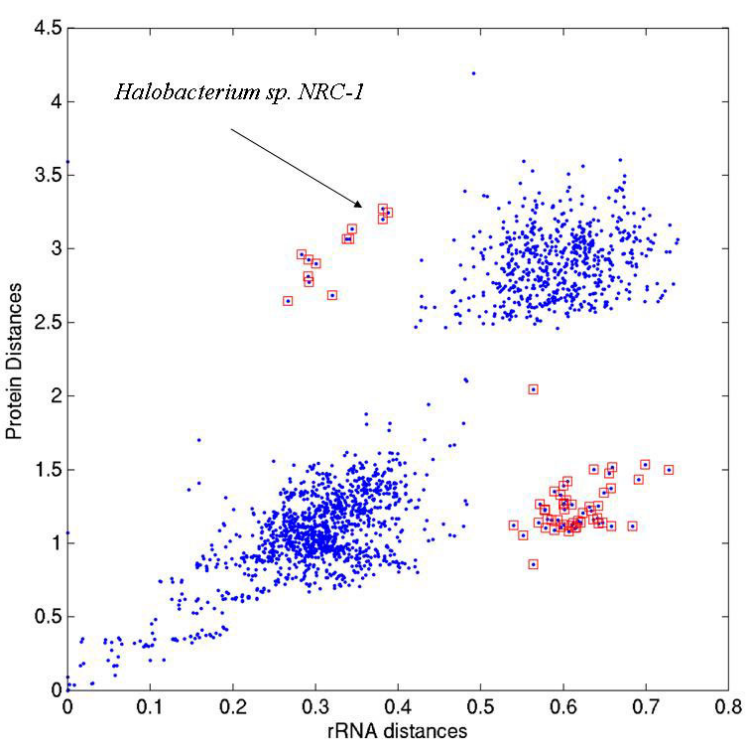
**Comparison of 16S rRNA distances with protein-protein distances in orthologous family of leucyl-tRNA synthetases (COG0495)**. The points indicated by red squares correspond to distances of VNG2223G (from *Halobacterium sp. NRC-1*) with other proteins from this family.

The correlation plot of leucyl-tRNA synthetases is shown in Fig. 4. Recently, a detailed analysis of this family has shown that leucyl-tRNA synthetase has been horizontally transferred from bacteria to the archaeon, *Halobacterium sp. NRC-1 *[21]. It is clear from the Fig. 4 that the points corresponding to *Halobacterium sp. NRC-1 *(COG protein ID, VNG2223G) stand out in the plot, and they were picked up by our <CDISS> measure.

### Percentage of transfer in different bacteria

After verifying our new method on previously characterized HGTs, we applied the method to 1965 protein families available in the COG database. These families show a correlation coefficient above 0.3 (see Methods) between protein and rRNA distances. Among these COGs, 4183 proteins were predicted as horizontally acquired. We could also rank the genomes according to the percentage of horizontally acquired genes [see Additional file 2]. Interestingly, for the top four of these genomes we find independent evidence in the literature supporting this high rate of HGT [1,27,28]. We observed the highest percentage of gene acquisition in *Aquifex aeolicus *and *Methanosarcina acetivorans*. It was shown earlier that hyperthermophilic bacterium, *A. aeolicus*, has very high percentage of genes with archaeal origin [1]. The archaeon, *M. acetivorans*, was also previously predicted to have acquired ~30% of genes from bacterial lineage [28]. *Halobacterium sp. NRC-1 *and *Pyrococcus abyssi *showed the third and fourth highest percentage of HGT. The lowest percentage was found among *Lactococcus lactis, Pasteurella multocida*, *Neisseria meningitidis, Streptococcus pyogenes, Yersinia pestis *and *Escherichia coli*. Previously, Koonin *et al*. [27,28] have also shown that *N. meningitidis and E. coli *display comparatively low percentage of horizontally acquired genes.

### Computing a core set of horizontally transferred genes

The 4183 proteins discussed above were picked up based on the <CDISS> measure alone. Simulation results and comparison with known biological examples indicated that the <CDISS> measure picked up horizontally transferred genes in majority of the cases (Table 1). However, to reduce the possibility of false-positives, we further short-listed 171 proteins which show very high similarity with a distant bacteria and hence probably have undergone HGT. These 171 genes have passed three stringent criteria of HGT, *viz*. outlier detection, high sequence similarity with distant sequence and likelihood tests confirming anomalies in protein sequence trees. The Methods Section describes the details of this filtering. We have high confidence that these 171 genes have indeed undergone horizontal gene transfer. We further divided these genes based on the type of transfer. Among these 171 genes, 118 genes have been transferred between archaea and bacteria. The remaining 53 genes have been transferred between different bacteria.

### Genes transferred between archaea and bacteria

The 118 genes, exchanged between archaea and bacteria [see Additional file 3] were further divided into those, which have been transferred from archaea to bacteria and from bacteria to archaea. Surprisingly around 74% of these transfers were from bacteria to archaea. A large majority of transfers were to *Methanosarcina acetivorans *(35.6%) and *Halobacterium sp. NRC*-1 (18.6%). Among these archaeal genes, there were genes involved in pathways such as citric acid cycle, proline biosynthesis and glycerol metabolism.

Among, the remaining 26% of genes, transferred from archaea to bacteria, the majority were acquired by Firmicutes (9.3%), *viz*., *Clostridium acetobutylicum *(7.6%) and *Bacillus *species (1.7%) and thermophiles (11.9%) *viz. Aquifex aeolicus *(3.4%), *Thermotoga maritima *(5.9%) and *Deinococcus radiodurans *(2.5%).

### Horizontal gene transfer between different bacterial phyla

A similar analysis was carried out on 53 genes [see Additional file 4] where transfer between different bacterial phyla (rRNA distance > 0.3) is observed. The majority of genes are acquired by the thermophiles (54.7%). The highest number of genes was gained by *Aquifex aeolicus *(37.7%), followed by *Deinococcus radiodurans *(13.2%) and *Thermotoga maritime *(3.8%).

### Functional characteristics of transferred genes

As mentioned earlier, the transferred genes are fixed in genomes probably because they confer an evolutionary advantage to the host. Different groups have analyzed functional categories of horizontally transferred genes and have reported conflicting results. Jain *et al*. [29] suggested that HGT may have preferentially occurred among cell growth maintenance and metabolism-related genes rather than among genes involved in transcription and translation. In a separate study, Nakamura *et al*. [30] found that horizontally transferred genes are biased towards cell surface, DNA binding and pathogenicity related functions. However, Choi and Kim [31] did not find any strong preference for a particular functional category.

**Figure 5:**
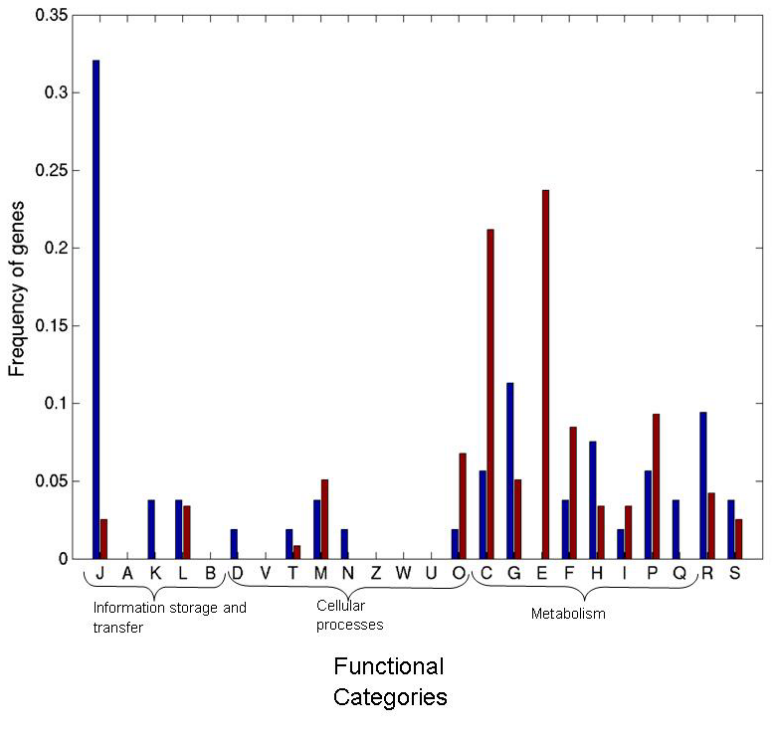
**Distribution of recent transfer events in COG functional categories**. Red bars indicate transfer between bacteria and archaea. Blue bars indicate transfer between different bacteria. The one letter code corresponds to following functional categories. **J **Translation, ribosomal structure and biogenesis; **A **RNA processing and modification; **K **Transcription; **L **Replication, recombination and repair; **B **Chromatin structure and dynamics; **D **Cell cycle control, cell division, chromosome partitioning; **Y **Nuclear structure; **V **Defense mechanisms; **T **Signal transduction mechanisms; **M **Cell wall/membrane/envelope biogenesis; **N **Cell motility; **Z **Cytoskeleton; **W **Extracellular structures; **U **Intracellular trafficking, secretion, and vesicular transport; **O **Posttranslational modification, protein turnover, chaperones; **C **Energy production and conversion; **G **Carbohydrate transport and metabolism; **E **Amino acid transport and metabolism; **F **Nucleotide transport and metabolism; **H **Coenzyme transport and metabolism; **I **Lipid transport and metabolism; **P **Inorganic ion transport and metabolism; **Q **Secondary metabolites biosynthesis, transport and catabolism; **R **General function prediction only; **S **Unknown function

Therefore, we analyzed the functional categories of the 171 gene candidates, which most probably have been horizontally acquired – 118 transferred between archaea and bacteria along with 53 transferred between bacterial phyla. The analysis of their functional categories, as described in COG, clearly showed distinct functional preferences among different groups of transferred genes. The genes transferred from bacteria to archaea were very strongly enriched in metabolism related genes (Fig. 5). On the other hand, the genes, which were transferred within the bacterial domain, were more populated with translation related genes (COG category: J) than with metabolic genes (Fig. 5).

## Discussion

In this paper we proposed, a simple framework for detection of horizontal gene transfer. The general assumption behind this framework is that genomic distances correlate with distances of proteins, evolving at constant rate. The procedure is based on detecting anomalies in correlation of genomic and protein distances. In contrast to a related previous study [13], the present approach is simpler and defines a single parameter, Cook's distance, for detection of HGT. This score performs well on simulated data as well as on known biological examples. This approach can be successfully applied to a much larger dataset than the dataset used in the earlier study [13]. Although using present day proteins, our simulation results show that Cook's distance measure can be used to detect transfers between distant as well as closely related bacteria (Fig. 2). Robust support for each of the predictions can be obtained only by detailed individual analysis. But our correct prediction of the previously known HGT events in aminoacyl-tRNA synthetases and the prediction of high frequency of HGTs in *A. aeolicus *further support the overall success of this method.

As mentioned in our simulation results, we chose a <CDISS> cut-off such that the false positive rate was restricted to only 5%. We chose this stricter and conservative cut-off to reduce false positives but this makes the method less sensitive to detection of transfers within closely related bacteria. In particular, we can detect only 40% of transfers within bacteria belonging to closely related genera (16S rRNA distance < 0.1 nucleotide substitutions per site). In addition, the method relies on the availability of orthologous protein sequences from different bacteria. The method cannot be applied to sequences where such information is either unavailable or insufficiently available. Hence acquisition of genes belonging to a flexible gene pool, like mobile elements or prophages, is difficult to detect using this method. Also, important requirement for this method is that the protein family under study should evolve at a constant rate. Protein families, which do not adhere to this criterion, cannot be analyzed by this method. Another simplification lies in the use of 16S rRNA sequences to calculate intergenomic distances. While generally useful for delineation of a species tree, in rare cases even the 16S rRNA distances show anomalies. In such cases, usage of other measures of intergenomic distances may be required.

In spite of the above limitations, the principle behind this approach offers certain inherent advantages over other methods. The predictions of similarity based methods, as recently proposed by Podell and Gaasterland [8], depend on fine tuning of many factors like filter threshold, definition of 'self' species or ranking of BLAST hits. On the other hand, our simplification of Novichkov *et al*.'s original approach depends only on a single robust parameter <CDISS>. In contrasts to Novichkov et al.'s approach, our method is not limited to detecting XGDs but also transfer of novel genes. Without the need to compute and compare phylogenetic trees, this approach is computationally less demanding and can be applied to large-scale datasets. Unlike similarity-based methods [1,8,9], which depend on large-scale genomic information, this approach utilizes sequences from a single orthologous protein family. Also, it is not sensitive to unavailability of certain gene sequences from a given set of organisms. In contrast to composition based methods, our approach uses protein sequences and hence our results are not influenced by nucleotide compositional variations due to codon bias or positive selection. It is difficult to establish the direction of horizontal transfer using similarity based methods. This, too, is remedied by the <CDISS> measure that identifies only those genes, which have been horizontally acquired (Fig. 1).

We have also derived a dataset of 171 horizontally transferred genes. Detailed analyses of this selected set of genes, which are involved in HGTs, give some interesting insights. Surprisingly, the transfers between bacteria and archaea mainly consist of gene transfer from bacteria to archaea rather than the other way around. We also find that the majority of these recent HGT events have occurred among metabolic genes. Archaea, which obtained genes from bacteria, mainly involve *M. acetivorans *and *Halobacterium sp. NRC-1*. This observation is in accordance with the fact that *Methanosarcinaceae *are metabolically as well as physiologically very versatile and exist in extensively different environments [32]. Acquiring new metabolic genes like those involved in proline biosynthesis possibly helps in coping better with the diverse ecological environment. The other archaeon, *Halobacterium sp. NRC-1 *also seems to have obtained important enzymes like those involved in the TCA cycle (fumarase) and Glycerol biosynthesis (glycerol dehydrogenase). It is postulated that moderate thermophiles like *Halobacterium sp. NRC-1 *share their habitats with multiple bacterial species and hence appear to possess a much greater number of acquired bacterial genes than other archaeal species [27]. It is interesting to note that *Halobacterium *is one of the few archaea, which possess TCA cycle.

Fewer genes have been transferred from archaea to bacteria than *vice versa*. These genes are mainly acquired by thermophilic bacteria – *A. aeolicus*, *T. maritima *and *D. radiodurans*. This is in agreement with previous suggestion that these thermophilic bacteria probably acquired genes from archaea, which are also hyperthermophiles [1,27]. Interestingly though, the number of genes acquired by these thermophiles from other bacteria is much more than those obtained from archaea.

Another interesting observation is the higher frequency of transfer between bacterial genomes of translational genes as compared to metabolic genes. Although, a number of HGT events in the translational proteins have been reported earlier [33-35], higher propensity of these genes in HGT compared to any other functional category has not been reported. There is a striking difference in frequencies of functional categories of genes transferred within bacterial phyla as compared to the genes transferred between archaea and bacteria. Such differences in the functional categories between interphyletic and intraphyletic transfers are only reported in cyanobacterial genome analysis [36]. This difference in transfer frequencies can be explained based on the 'Complexity Hypothesis' proposed by Jain *et al*. [29]. Most of the translational category proteins are part of a multi-protein machinery. Replacement of a single protein in this machinery by a distant ortholog is probably hazardous. This may explain our observation of the low frequency of horizontally transferred translational proteins between archaea and bacteria as compared to the interbacterial transfers.

## Conclusion

In this article, expanding on a previously proposed method by Novichkov *et al*., we proposed a simplified approach to detection of horizontal gene transfer. The simplification helped us in applying the method to a much larger dataset. Using simulations as well as already known examples of horizontal gene transfer we also showed that the method performed well. Our simulation indicated that the method could detect transfers much more efficiently than previous method. This increase in efficiency is due to the different design of the two methods. Novichkov et al.'s method is designed to detect only XGDs while our method can detect XGDs as well novel gene acquisitions. Our method is more efficient in detecting HGT between distantly related bacteria than between closely related bacteria. Although not devoid of limitations, our method has added advantages over other methods proposed previously.

We further filtered our predictions using stringent sequence similarities as well as likelihood tests. This led to a dataset of 171 genes, which with high confidence had undergone horizontal gene transfer. We hope that these 171 genes will be useful in future analysis. Our analysis of 171 genes provided interesting insights into nature of horizontal transfers. The majority of transfers between archaea and bacteria have occurred in the direction from bacteria to archaea rather than the other way round.

There is a disagreement over functional properties of horizontally transferred genes. Hence we carried out analysis of functional properties of these genes. Genes transferred between the archaea and bacteria are mostly metabolic genes. On the other hand, genes transferred between bacterial phyla are mainly involved in translation. Our finding indicated that functional property of a transferred gene probably depends on phylogenetic distance between the acceptor and donor bacteria.

## Methods

### Protein and 16S rRNA sequences

For this study we used the Clusters of Orthologous Groups (COGs) database [14]. All the bacterial protein sequences corresponding to 4569 COGs were downloaded from NCBI ftp-site . We performed the analyses on microbial protein sequences from 63 bacterial and archaeal genomes [see Additional file 5]. For this study, we have used 16S rRNA distances to represent intergenomic distances. The 16S rRNA sequences were obtained from the NCBI website. Availability and analysis of large number of prokaryotic genomes have shown that evolutionary relation between prokaryotes is well represented by 16S rRNA sequences [19]. The tree based on prokaryotic 16S rRNA sequences used in this analysis is very similar to recently published tree [37].

### Multiple alignments and distance calculation

Multiple alignments of protein sequences in each COG were produced using CLUSTALW 1.83 [38]. Default input parameters were used for these alignments. The distances between rRNA and protein sequences were calculated using DNAdist and protdist program, provided by PHYLIP package [39], respectively. DNAdist was run using F84 model and protdist was run using Dayhoff's PAM matrix model. For each COG with N proteins, all (N+1)*N/2 pairwise distances between protein and between 16S rRNA were measured. A linear regression analysis between these protein distances and corresponding 16S rRNA distances is carried out. Only 1965 COG families, which show a correlation above r > 0.3 (p-val < 10^-5^) and which have more than 8 protein members were used for further analysis. This step was used to make sure that the protein-protein distances in the family are generally proportional to corresponding distances between 16S rRNAs.

### Cook's distance (CDISS)

Cook's distance is used to detect outliers in a correlation [15,16]. In this study, the CDISS measure was used to detect a protein-protein distance (Y_j_) not in agreement with the corresponding rRNA-rRNA distance (X_j_). A linear regression relation between X_j _and Y_j _is given by:

(1) $${\hat{Y}}_{j}=a+bX_{j}$$

where ${\hat{\text{Y}}}_{\text{j}}$ is the predicted value for Y_j _based on linear regression equation 1.

For the i^th ^distance pair (X_i_, Yi), the CDISS_i _value is calculated as follows:

(2) $$CDISS_{i}=\frac{\sum_{j\neq i}{\left({\hat{Y}}_{j}-{\hat{Y}}_{j}(i)\right)}^{2}}{p\times MSE}$$

where,

${\hat{Y}}_{j}$(*i*) = Newly predicted value of *Y*_*j *_using new regression parameters *a*(*i*) and *b*(*i*) calculated after deleting the i^th ^observation (*X*_*i*_, *Y*_*i*_).

p = Number of regression parameters (i.e.,2, constant 'a' and slope 'b' as in equation 1) 

MSE = Mean squared error w.r.t. regression line ${\hat{Y}}_{j}$ = *a *+ *bX*_*j*_

Here, the mean <CDISS> value over all the distances involving a given protein was used as a measure of its deviation from the expected phylogenetic relation. A cut-off of 2/D (D = number of protein-protein distances, (N+1)*N/2, in given COG family) was used for prediction of horizontally transferred genes. In our simulation (see Results) 90% of True Positives and 5% of false positives are observed to be above this cut-off.

### Core set of horizontally transferred genes

Each candidate protein predicted to be horizontally transferred, was aligned with every other protein sequence in the given protein family. All pairwise alignments were done with the help of the EMBOSS package using the Needleman-Wunsch algorithm [40]. All the HGT candidate proteins, which show high sequence identity (at least 40%) with a distant protein, were selected. Based on this criterion we could select 257 such proteins. Archaeal proteins with best match with a bacterial protein were considered as acquired from bacteria. Similarly, bacterial proteins with best match from archaea were considered as acquired from archaeal domain. All the transfers between bacteria, with 16S rRNA distance > 0.3 and belonging to different phyla, are noted as transfers within bacterial domain. Only 171 genes, which passed the likelihood tests described below, were short-listed.

### Likelihood calculation and tree comparisons

For likelihood calculations, we divided a COG family into the following groups-Archaea, Actinobacteria, Firmicutes, γ-proteobacteria, α-proteobacteria, β-proteobacteria, Chlamydiae-Spirochaetes and rest of the bacteria (Aquificae, Thermotogae, Cyanobacteria, Deinococcus-Thermus, Fusobacteria). From a given COG, a set of sequences, comprising subgroup of bacteria to which transferred gene belongs and subgroup of bacteria to which donor gene belongs, was selected. For each such set, we built a protein tree and a 16S rRNA tree using the Tree-Puzzle package [41]. Protein trees were built using VT model and 16S rRNA tree were built using HKY model. We compared the likelihood of protein sequences to have evolved according to protein tree and 16S rRNA tree using Shimodaira-Hasegawa test [42], one-sided Kishino-Hasegawa test [43] and expected likelihood weight test [44]. The likelihood calculations and statistical tests were also carried out using the Tree-Puzzle package. For a given group of bacteria, we checked whether the 16S rRNA tree could be rejected at a 5% significance level. In case of a HGT, the likelihood of the protein family to follow the 16S rRNA tree is significantly lower than to follow the protein tree. For sake of clarity and ease of interpretation, the calculations were carried out on smaller groups rather than entire COG family. Some families had more than one incident of HGT. In such cases, results could be much easily understood if the COG family was divided into individual groups of bacteria. All the likelihood based tree comparisons are provided [see Additional file 6].

In certain cases, where the number of bacteria in a given group was less than 4, we could not carry out above calculation.

Among the 257 sequences, for 171 sequences the 16S rRNA tree could be rejected at 5% significance level. These 171 sequences are used for further analysis.

These 171 sequences thus passed three criteria of HGT, namely Cook's distance, similarity criteria and likelihood-based tests.

The functional categories of these short-listed proteins were obtained from the COG database.

## Authors' contributions

AK and MV conceived the idea. AK performed the analysis and drafted the versions of the manuscript. MV contributed with discussion of the draft versions and critical review. All authors read and approved the manuscript.

## Supplementary Material

Additional file 1 **List of COGs. List of COGs used in this study.** Click here for file

Additional file 2 **Percentage of HGT. Percentage of horizontally acquired genes in different genomes.** Click here for file

Additional file 3 **Archaeal-Bacterial HGT. HGT between Archaea and Bacteria.** Click here for file

Additional file 4 **Intra-phyletic transfer. HGT among different bacterial phyla.** Click here for file

Additional file 5 **Name of organisms in this study. Name, abbreviation and classification of organisms, whose genomic sequences were used in this study.** Click here for file

Additional file 6 **Likelihood based tree comparisons. All likelihood based tree comparison outputs are provided.** Click here for file
