# Supplementary material for: Horizontal Gene Transfers in prokaryotes show differential preferences for metabolic and translational genes
Source: BMC Evol Biol. 2009 Jan 10;9:9. doi: 10.1186/1471-2148-9-9 (PMC2651853; doi:10.1186/1471-2148-9-9)
Supplement: Additional file 3 — Archaeal-Bacterial HGT. HGT between Archaea and Bacteria. [file 1471-2148-9-9-S3.html]

|  |  |  |  |  |  |  |  |
| --- | --- | --- | --- | --- | --- | --- | --- |
| Transferred Gene | Acceptor | Acceptor Type | Donor Gene | Donor | Donor Type | %id | %sm |
| MA3918 | Mac | Archaea | lin1551 | Lin | Firmicutes | 44.9 | 66.6 |
| MA4100 | Mac | Archaea | sll0373 | Syn | Cyanobacteria | 47.3 | 63.9 |
| CAC1432 | Cac | Firmicutes | MA1831 | Mac | Archaea | 62.1 | 74.1 |
| MA1831 | Mac | Archaea | CAC1432 | Cac | Firmicutes | 62.1 | 74.1 |
| VNG0632G | Hbs | Archaea | SMc04002 | Sme | alpha | 42.1 | 49.5 |
| VNG1883G | Hbs | Archaea | CC2913 | Ccr | alpha | 40.3 | 51.1 |
| MJ0680 | Mja | Archaea | BH2502 | Bha | Firmicutes | 44.5 | 61 |
| MA2434 | Mac | Archaea | SA1907 | Sau | Firmicutes | 47.8 | 64.4 |
| MA3790 | Mac | Archaea | all2315 | Nos | Cyanobacteria | 65.4 | 76.1 |
| CAC3173 | Cac | Firmicutes | PAB0891 | Pab | Archaea | 60.2 | 79.8 |
| DR1610 | Dra | Deinococcus-Thermus | AF2199 | Afu | Archaea | 43.5 | 60.8 |
| CAC3172 | Cac | Firmicutes | PAB0892 | Pab | Archaea | 66.5 | 78.7 |
| DR1614 | Dra | Deinococcus-Thermus | PAB0288 | Pab | Archaea | 42 | 59.1 |
| MA1919 | Mac | Archaea | SP0019 | Spn | Firmicutes | 41.7 | 60 |
| MA4118 | Mac | Archaea | BH4028 | Bha | Firmicutes | 47.1 | 66 |
| VNG1089G | Hbs | Archaea | SMc00643 | Sme | alpha | 44.5 | 60.6 |
| MA3520 | Mac | Archaea | CAC2264 | Cac | Firmicutes | 62.6 | 77.1 |
| VNG1414G | Hbs | Archaea | CAC2264 | Cac | Firmicutes | 52.8 | 68.4 |
| MA1001 | Mac | Archaea | slr0018 | Syn | Cyanobacteria | 53 | 68.1 |
| VNG1356G | Hbs | Archaea | fumC | Eco | gamma | 52.2 | 66.9 |
| MA0219 | Mac | Archaea | lin0575 | Lin | Firmicutes | 52.6 | 67.5 |
| MA2991 | Mac | Archaea | TM0138 | Tma | Thermotogae | 62.9 | 76.4 |
| MA2300\_1 | Mac | Archaea | mlr1573\_1 | Mlo | alpha | 51.2 | 66.6 |
| MA4048 | Mac | Archaea | aq\_298 | Aae | Aquificae | 57.7 | 74.6 |
| VNG2072G | Hbs | Archaea | BS\_serS | Bsu | Firmicutes | 45 | 62.2 |
| MA4014 | Mac | Archaea | all3970 | Nos | Cyanobacteria | 44.6 | 60.5 |
| MA0523 | Mac | Archaea | BH2369 | Bha | Firmicutes | 45.1 | 62.3 |
| BS\_ansA | Bsu | Firmicutes | PAB2294 | Pab | Archaea | 40.1 | 60.8 |
| MTH818 | Mth | Archaea | BH1352 | Bha | Firmicutes | 52.6 | 64.7 |
| VNG1859G | Hbs | Archaea | BH1352 | Bha | Firmicutes | 44.5 | 58.5 |
| RSc2773 | Rso | Proteobacteria | VNG1673G | Hbs | Archaea | 41.1 | 58.2 |
| SSO2093 | Sso | Archaea | all0168 | Nos | Cyanobacteria | 41.5 | 56.9 |
| AF1485 | Afu | Archaea | BS\_tgt | Bsu | Firmicutes | 44.1 | 61.6 |
| MA4102 | Mac | Archaea | lin0414 | Lin | Firmicutes | 48.3 | 67.2 |
| CAC0254 | Cac | Firmicutes | MTH1561 | Mth | Archaea | 53.2 | 70.3 |
| CAC0255 | Cac | Firmicutes | MA3897 | Mac | Archaea | 48.5 | 65.4 |
| MA3916 | Mac | Archaea | TM0403 | Tma | Thermotogae | 48.8 | 61.4 |
| VNG6270G | Hbs | Archaea | STM4108 | Sty | gamma | 46.9 | 61.9 |
| MA0972 | Mac | Archaea | mlr6940 | Mlo | alpha | 73.8 | 81.5 |
| RSc0775 | Rso | Proteobacteria | MA0972 | Mac | Archaea | 51 | 54.9 |
| FN0129 | Fnu | Fusobacteria | MA1228 | Mac | Archaea | 62.3 | 78.4 |
| PAE1808 | Pya | Archaea | SPy0285 | Spy | Firmicutes | 43.4 | 57.7 |
| VNG0524G | Hbs | Archaea | L34806 | Lla | Firmicutes | 46.2 | 61.5 |
| VNG0715G | Hbs | Archaea | all0982 | Nos | Cyanobacteria | 55.8 | 68.4 |
| MA0387 | Mac | Archaea | Cgl1400 | Cgl | Actinobacteria | 42.5 | 57.8 |
| MA3457 | Mac | Archaea | BH0570 | Bha | Firmicutes | 41.5 | 60.6 |
| MA0631 | Mac | Archaea | aq\_2200 | Aae | Aquificae | 55.8 | 75 |
| MA1862 | Mac | Archaea | CAC0456 | Cac | Firmicutes | 52.1 | 72.6 |
| VNG1967G | Hbs | Archaea | glpK | Eco | gamma | 55 | 70.5 |
| aq\_1499 | Aae | Aquificae | TVN0061 | Tvo | Archaea | 52.6 | 62.9 |
| MA2436 | Mac | Archaea | FN0363 | Fnu | Fusobacteria | 41.8 | 60.4 |
| CAC1632 | Cac | Firmicutes | PH1905 | Pho | Archaea | 43.1 | 60.8 |
| MA1503 | Mac | Archaea | sll0522 | Syn | Cyanobacteria | 51 | 73.1 |
| VNG0643G | Hbs | Archaea | DR1495 | Dra | Deinococcus-Thermus | 49.5 | 66 |
| MA1495 | Mac | Archaea | slr1279 | Syn | Cyanobacteria | 40.3 | 57.3 |
| BH0739 | Bha | Firmicutes | VNG2193Gm | Hbs | Archaea | 40.3 | 56.3 |
| MA1499 | Mac | Archaea | alr0223 | Nos | Cyanobacteria | 40.7 | 58.8 |
| VNG0639G | Hbs | Archaea | BMEI1151 | Bme | alpha | 41.6 | 60.9 |
| MA4572\_1 | Mac | Archaea | lin2477\_1 | Lin | Firmicutes | 41.4 | 61.4 |
| MA0403 | Mac | Archaea | Cj1548c | Cje | Proteobacteria | 43.4 | 58.2 |
| MTH631 | Mth | Archaea | all4713 | Nos | Cyanobacteria | 56.4 | 70.1 |
| MA2837 | Mac | Archaea | VC1422 | Vch | gamma | 59.9 | 74.7 |
| VNG6264G | Hbs | Archaea | BH1394 | Bha | Firmicutes | 43 | 58.8 |
| MA1910 | Mac | Archaea | SPy2004 | Spy | Firmicutes | 41.6 | 70.3 |
| AF0981 | Afu | Archaea | BS\_opuCA | Bsu | Firmicutes | 42.8 | 59.8 |
| CAC0116 | Cac | Firmicutes | MJ0728 | Mja | Archaea | 49.3 | 68.1 |
| CAC2498 | Cac | Firmicutes | AF1849 | Afu | Archaea | 51.6 | 70.2 |
| MJ0765 | Mja | Archaea | CAC2750 | Cac | Firmicutes | 61 | 74.1 |
| TP0528 | Tpa | Chlam-Spir | PH1974 | Pho | Archaea | 55.4 | 72.2 |
| DRA0031 | Dra | Deinococcus-Thermus | PH0413 | Pho | Archaea | 41.5 | 61.5 |
| MTH1791 | Mth | Archaea | CAC2333 | Cac | Firmicutes | 67.8 | 83.2 |
| MA4459 | Mac | Archaea | BH3652 | Bha | Firmicutes | 51.2 | 67.4 |
| PAB0771 | Pab | Archaea | CAC2250 | Cac | Firmicutes | 54.5 | 72.6 |
| MA4339 | Mac | Archaea | aq\_1359 | Aae | Aquificae | 41.4 | 61.9 |
| VNG2220G | Hbs | Archaea | lin1047 | Lin | Firmicutes | 48.4 | 65.5 |
| VCA0723 | Vch | gamma | MK0355 | Mka | Archaea | 40.3 | 55.8 |
| MA1855 | Mac | Archaea | BH0429 | Bha | Firmicutes | 53.3 | 72 |
| MA3262 | Mac | Archaea | CAC0611 | Cac | Firmicutes | 52.7 | 73.6 |
| TM0087 | Tma | Thermotogae | SSO0066 | Sso | Archaea | 44.6 | 62.5 |
| PAE2453 | Pya | Archaea | BS\_tdk | Bsu | Firmicutes | 45.5 | 59.1 |
| VNG1515G | Hbs | Archaea | CAC2887 | Cac | Firmicutes | 49.5 | 68.7 |
| MA2630 | Mac | Archaea | ZyiaY | EcZ | gamma | 48.8 | 66.5 |
| MA1152 | Mac | Archaea | BH3783 | Bha | Firmicutes | 42.3 | 60.7 |
| MA3979 | Mac | Archaea | FN1866 | Fnu | Fusobacteria | 53.2 | 69 |
| AF0403 | Afu | Archaea | SA1722 | Sau | Firmicutes | 40.1 | 57.9 |
| MA2664 | Mac | Archaea | SPy0330 | Spy | Firmicutes | 54.5 | 72.2 |
| MA1905 | Mac | Archaea | FN0221 | Fnu | Fusobacteria | 54.7 | 68.8 |
| MA1979 | Mac | Archaea | BH3032 | Bha | Firmicutes | 49.6 | 69.4 |
| VNG6177G | Hbs | Archaea | mll3130 | Mlo | alpha | 50.2 | 68 |
| MA2502 | Mac | Archaea | all2088 | Nos | Cyanobacteria | 56.1 | 67.2 |
| UU221 | Uur | Mollicutes | TVN0089 | Tvo | Archaea | 40.7 | 62.5 |
| VNG2393G | Hbs | Archaea | Rv3117 | Mtu | Actinobacteria | 53.9 | 64.1 |
| MA3261 | Mac | Archaea | BH3162 | Bha | Firmicutes | 44.9 | 58 |
| VNG2021C\_1 | Hbs | Archaea | lin2218 | Lin | Firmicutes | 44.1 | 62.1 |
| PAE2696 | Pya | Archaea | BH1069 | Bha | Firmicutes | 58.1 | 72.3 |
| VNG2085G | Hbs | Archaea | all8090 | Nos | Cyanobacteria | 42 | 55.2 |
| VNG6281G | Hbs | Archaea | TM1276 | Tma | Thermotogae | 49.4 | 61.7 |
| CAC2001 | Cac | Firmicutes | PAB0718 | Pab | Archaea | 40.4 | 59 |
| VNG1532G | Hbs | Archaea | Rv3285 | Mtu | Actinobacteria | 46.7 | 61 |
| AF0209 | Afu | Archaea | aq\_1612 | Aae | Aquificae | 51.4 | 67.6 |
| MK1422 | Mka | Archaea | aq\_916 | Aae | Aquificae | 47.6 | 62.2 |
| PH0882 | Pho | Archaea | TM1417 | Tma | Thermotogae | 55.1 | 73.9 |
| MTH473 | Mth | Archaea | lin0435 | Lin | Firmicutes | 55 | 69.9 |
| AF0950 | Afu | Archaea | TM0396 | Tma | Thermotogae | 41.5 | 60.4 |
| MTH1453\_2 | Mth | Archaea | TM1172 | Tma | Thermotogae | 62 | 77.8 |
| PAB1707 | Pab | Archaea | TM1172 | Tma | Thermotogae | 49.8 | 68.4 |
| MTH1549\_1 | Mth | Archaea | TM0010\_1 | Tma | Thermotogae | 50.5 | 68.7 |
| MTH1548 | Mth | Archaea | TM1424 | Tma | Thermotogae | 40.2 | 59.2 |
| MJ1477 | Mja | Archaea | TM1410 | Tma | Thermotogae | 42.6 | 58.5 |
| TM0292 | Tma | Thermotogae | AF1761 | Afu | Archaea | 63.7 | 78.6 |
| TM0555 | Tma | Thermotogae | PAB0892 | Pab | Archaea | 60.7 | 79.2 |
| aq\_1398 | Aae | Aquificae | PAB0892 | Pab | Archaea | 50.7 | 60.7 |
| TM0398 | Tma | Thermotogae | AF0952 | Afu | Archaea | 52.1 | 71.9 |
| TM1417 | Tma | Thermotogae | PH0882 | Pho | Archaea | 55.1 | 73.9 |
| TM0807 | Tma | Thermotogae | MJ0736 | Mja | Archaea | 74.3 | 85.1 |
| aq\_890 | Aae | Aquificae | MJ0403 | Mja | Archaea | 43.8 | 58.7 |
| TM0787 | Tma | Thermotogae | PAB0536 | Pab | Archaea | 51 | 70.8 |
| aq\_1790 | Aae | Aquificae | MK0954 | Mka | Archaea | 60.6 | 73.7 |
