# Supplementary material for: Horizontal Gene Transfers in prokaryotes show differential preferences for metabolic and translational genes
Source: BMC Evol Biol. 2009 Jan 10;9:9. doi: 10.1186/1471-2148-9-9 (PMC2651853; doi:10.1186/1471-2148-9-9)
Supplement: Additional file 4 — Intra-phyletic transfer. HGT among different bacterial phyla. [file 1471-2148-9-9-S4.html]

|  |  |  |  |  |  |  |  |
| --- | --- | --- | --- | --- | --- | --- | --- |
| Transferred Gene ID | Acceptor | Acceptor Type | Donor Gene ID | Donor | Donor Type | %id | %sm |
| CAC2979 | Cac | Firmicutes | DR1055 | Dra | Deinococcus-Thermus | 45.4 | 63 |
| Cj0514 | Cje | Proteobacteria | alr2475 | Nos | Cyanobacteria | 49.1 | 67.3 |
| MYPU\_2680 | Mpu | Mollicutes | FN0360 | Fnu | Fusobacteria | 56.6 | 69.8 |
| DR2195 | Dra | Deinococcus-Thermus | MT1370 | MtC | Actinobacteria | 40.6 | 56.4 |
| CAC3038 | Cac | Firmicutes | BB0833 | Bbu | Chlam-Spir | 41.4 | 61.7 |
| AGpA25 | Atu | alpha | DRC0006 | Dra | Deinococcus-Thermus | 40.2 | 59 |
| aq\_011 | Aae | Aquificae | Rv0702 | Mtu | Actinobacteria | 41.1 | 54.5 |
| aq\_074 | Aae | Aquificae | TP0210 | Tpa | Chlam-Spir | 65.9 | 73.8 |
| aq\_1878 | Aae | Aquificae | SPy1931 | Spy | Firmicutes | 43.5 | 55.8 |
| aq\_350\_1 | Aae | Aquificae | VC2269\_1 | Vch | gamma | 62.1 | 73.5 |
| TM1493 | Tma | Thermotogae | BS\_rplP | Bsu | Firmicutes | 66.7 | 79.9 |
| aq\_070 | Aae | Aquificae | mlr0325 | Mlo | alpha | 43.1 | 58.9 |
| DR2524 | Dra | Deinococcus-Thermus | L0422 | Lla | Firmicutes | 40.7 | 53.1 |
| aq\_579 | Aae | Aquificae | NMB0110 | Nme | Proteobacteria | 43.9 | 59.5 |
| aq\_2042 | Aae | Aquificae | NMA1534 | NmA | Proteobacteria | 41 | 57.7 |
| BB0152 | Bbu | Chlam-Spir | YPO2627 | Ype | gamma | 61.3 | 78.4 |
| Cgl0114 | Cgl | Actinobacteria | TM1077 | Tma | Thermotogae | 47.8 | 59 |
| BB0402 | Bbu | Chlam-Spir | ML1553 | Mle | Actinobacteria | 44.8 | 65.3 |
| DR1266 | Dra | Deinococcus-Thermus | ML1553 | Mle | Actinobacteria | 48.8 | 65 |
| ML1553 | Mle | Actinobacteria | DR1266 | Dra | Deinococcus-Thermus | 48.8 | 65 |
| PA0779 | Pae | gamma | CPn0027 | Cpn | Chlam-Spir | 48.4 | 66.7 |
| DR1674 | Dra | Deinococcus-Thermus | CAC0972 | Cac | Firmicutes | 48.7 | 66.3 |
| aq\_1224 | Aae | Aquificae | CAC2233 | Cac | Firmicutes | 44.5 | 62.3 |
| TM1473 | Tma | Thermotogae | CAC3105 | Cac | Firmicutes | 55 | 73.7 |
| aq\_2069 | Aae | Aquificae | CC0315 | Ccr | alpha | 48 | 63.4 |
| sll0992 | Syn | Cyanobacteria | ZyaiM | EcZ | gamma | 58.3 | 71.2 |
| aq\_924 | Aae | Aquificae | BS\_rph | Bsu | Firmicutes | 60.9 | 77 |
| aqq\_01 | Aae | Aquificae | CAC3737 | Cac | Firmicutes | 51.4 | 69.4 |
| aq\_1360 | Aae | Aquificae | AGc3796 | Atu | alpha | 44.7 | 63.1 |
| aq\_445 | Aae | Aquificae | RSc1980 | Rso | Proteobacteria | 52.4 | 69.5 |
| Cj1724c | Cje | Proteobacteria | aq\_931 | Aae | Aquificae | 54.1 | 72.2 |
| aq\_931 | Aae | Aquificae | Cj1724c | Cje | Proteobacteria | 54.1 | 72.2 |
| aq\_325 | Aae | Aquificae | lin1200 | Lin | Firmicutes | 41.9 | 58.5 |
| YPO1907 | Ype | gamma | BS\_dhbE | Bsu | Firmicutes | 47.3 | 62.3 |
| aq\_1531 | Aae | Aquificae | PA3212 | Pae | gamma | 41.7 | 59.1 |
| aq\_881 | Aae | Aquificae | RSc2221 | Rso | Proteobacteria | 50.5 | 64.4 |
| aq\_873 | Aae | Aquificae | PA5239 | Pae | gamma | 56.4 | 73.2 |
| aq\_337 | Aae | Aquificae | STM4404 | Sty | gamma | 44.9 | 62 |
| SPy0891 | Spy | Firmicutes | PM1944 | Pmu | gamma | 57.5 | 72.6 |
| DR0552 | Dra | Deinococcus-Thermus | SMc03867 | Sme | alpha | 47.2 | 56 |
| FN1473\_2 | Fnu | Fusobacteria | PM1708\_2 | Pmu | gamma | 75.9 | 87.5 |
| SPy0426 | Spy | Firmicutes | YPO2650 | Ype | gamma | 47 | 56.7 |
| CAC2942 | Cac | Firmicutes | BB0377 | Bbu | Chlam-Spir | 48.9 | 65.5 |
| yefM | Eco | gamma | ssr0761 | Syn | Cyanobacteria | 41.5 | 64.9 |
| BB0559 | Bbu | Chlam-Spir | PM0896 | Pmu | gamma | 46.4 | 64.9 |
| DR1898 | Dra | Deinococcus-Thermus | NMA1433 | NmA | Proteobacteria | 53.2 | 67.7 |
| aq\_1474 | Aae | Aquificae | PA3103 | Pae | gamma | 45.7 | 63 |
| Cgl0644 | Cgl | Actinobacteria | NMA1116 | NmA | Proteobacteria | 64.4 | 76.8 |
| SP0977\_1 | Spn | Firmicutes | HI1275\_1 | Hin | gamma | 59.1 | 75.3 |
| FN1060\_1 | Fnu | Fusobacteria | yfeS\_1 | Eco | gamma | 47.3 | 68.9 |
| YPO0854 | Ype | gamma | BH2021 | Bha | Firmicutes | 68.4 | 85.6 |
| DRA0291 | Dra | Deinococcus-Thermus | PA4128 | Pae | gamma | 66.3 | 74.6 |
| BS\_ycsN | Bsu | Firmicutes | YPO2375 | Ype | gamma | 52.3 | 68.5 |
